# Rigorous broadband study of the intrinsic ferromagnetic linewidth of monocrystalline garnet spheres

**DOI:** 10.1038/s41598-019-45699-7

**Published:** 2019-07-01

**Authors:** Adam Pacewicz, Jerzy Krupka, Bartlomiej Salski, Pavlo Aleshkevych, Pawel Kopyt

**Affiliations:** 1 Institute of Radioelectronics and Multimedia Technology, Warsaw University of Technology, Warsaw, 00-665 Poland; 2 Institute of Microelectronics and Optoelectronics, Warsaw University of Technology, Warsaw, 00-662 Poland; 3 Institute of Physics, Polish Academy of Sciences, Warsaw, 02-668 Poland

**Keywords:** Characterization and analytical techniques, Electronic and spintronic devices

## Abstract

This work demonstrates the first application of direct broadband (1 GHz–30 GHz) quality (*Q*) factor measurements of the uniform precession mode in magnetised garnet spheres for the accurate determination of the room-temperature intrinsic ferromagnetic linewidth (Δ*H*). The spheres were enclosed in a subwavelength cavity, so that the measured *Q*-factor depended mainly on their magnetic losses and the conduction losses of the cavity walls. The contribution of the latter is assessed by means of the recently proposed magnetic plasmon resonance model and has been found to be negligible. A total of 10 samples made from commercially available pure yttrium iron garnet (YIG) and gallium-substituted YIG have been measured, differing in diameter and/or saturation magnetisation *M*_*s*_. The dependence of the intrinsic Δ*H* on the internal magnetic field is found to have near-perfect linear dependence, which cannot be said about the typically studied extrinsic Δ*H* even at high frequencies. It is found that the difference between the two linewidths, which becomes significant at low frequencies, can be attributed to a geometric effect. Due to its fundamental nature, this work is applicable not only to magnetic material characterization, but also to the study of the origins of losses in magnetic materials.

## Introduction

The phenomenon of resonant absorption of radio frequency (RF) and microwave radiation in ferromagnetic materials has been heavily studied since the 1940s^1,2^. In recent years there has been a revived interest in yttrium iron garnet (YIG), as the material is finding applications in spintronics^3^ and quantum information processing^4–6^, notably due to its uniquely low magnetic losses. In particular, spherical YIG samples easily lend themselves to experimental studies primarily due to the large number of contained spins. This work is focused on the broadband characterization of intrinsic microwave loss in commercially available pure YIG as well as gallium-doped spheres by means of accurate measurements of their *Q*-factor. Appropriate doping of YIG has the effect of lowering the saturation magnetisation. The presented theoretical analysis and experiments are restricted to the mode of uniform precession, for which a rigorous electrodynamic model has been recently introduced and validated^7–9^. Such an electrodynamic approach has led to the discovery that the resonance observed in bulk ferromagnetic samples is a magnetic plasmon resonance (MPR), but has misfortunately been called the ferromagnetic resonance (FMR), which it is not. At FMR, the sample exhibits peak losses, while at MPR its permeability is negative and the losses are many orders of magnitude smaller than at FMR^7^.

The paper is organized as follows. First, fundamental properties of gyromagnetic materials are reviewed. In particular, the relationship between the intrinsic and extrinsic linewidths is elucidated. Second, the measurement setup and methods used to obtain the intrinsic ferromagnetic linewidth from the unloaded *Q*-factor of the spheres are described in detail. Third, experimental results of the linewidth are provided and discussed. Finally, an assessment of the contribution of conduction losses in the metal walls of the cavity is performed using the electrodynamic MPR model. The complete set of raw data has been made publicly available at https://osf.io/mwhdu/.

## Fundamental Properties of Gyromagnetic Materials

The intrinsic permeability of a ferromagnetic (gyrotropic) material saturated along the +*z* axis is a tensor of the following form^7^:

1 $$\bar{\mu }={\mu }_{0}[\begin{array}{ccc}\mu  & i\kappa  & 0\\ -i\kappa  & \mu  & 0\\ 0 & 0 & 1\end{array}].$$

Diagonal and off-diagonal components of the tensor, *μ* and *κ*, respectively, of a saturated ferromagnet in the low magnetic loss regime (Gilbert damping factor *α* ≪ 1) depend on the excitation frequency and internal magnetic field *H*_*int*_ and can be conveniently expressed as follows^7,10^:

2 $$\mu =1+\frac{{H}_{r}+i\alpha \hat{w}}{{H}_{r}^{2}-{\hat{w}}^{2}+2i\alpha {H}_{r}\hat{w}}=\mu ^{\prime} -i\mu ^{\prime\prime} ,$$

3 $$\kappa =\frac{\hat{w}}{{H}_{r}^{2}-{\hat{w}}^{2}+2i\alpha {H}_{r}\hat{w}}=\kappa ^{\prime} -i\kappa ^{\prime\prime} ,$$

where *H*_*r*_ = *H*_*int*_/*M*_*s*_ is the relative internal magnetic bias, $\hat{w}=\hat{f}/(\gamma {M}_{s})$ is the relative complex frequency $(\hat{f}=f+i\frac{f}{2{Q}_{0}})$, *M*_*s*_ is the saturation magnetisation of the sample, *γ* ≈ 2.8 MHzOe^−1^ is the gyromagnetic ratio, and *Q*_0_ is the unloaded *Q*-factor of the resonant system.

As any resonance, the MPR is characterized by a linewidth, in this case known as the ferromagnetic linewidth. Traditionally, its experimental determination consists in measuring the 3 dB power bandwidth either in the frequency domain (Δ*f*) for different values of the magnetic bias, or in the field domain (Δ*H*_*ext*_) for different excitation frequencies. The use of the subscript *extrinsic* is to emphasize that it is related to externally applied bias fields.

On the other hand, the intrinsic ferromagnetic linewidth Δ*H*_*int*_, related to the *internal* magnetic field, is understood as the full width at half maximum (FWHM) of *μ*″. The relationship between *α* and Δ*H*_*int*_ is the following^11^:

4 $$\alpha =\frac{{\rm{\Delta }}{H}_{int}}{2{H}_{int}}.$$

Based on the above intrinsic material properties, the MPR model provides a characteristic equation enabling the computation of complex resonant frequencies and *Q*-factors of a spherical gyromagnetic resonator enclosed in a concentric spherical perfectly conducting shield^7^.

According to scattering theory^12^ and approximate electrodynamic^13^ considerations, the relation between MPR frequency *f* and external magnetic field *H*_*ext*_ of a ferromagnetic sphere located in free space can be approximated by the formula:

5 $$f=\gamma ({H}_{ext}+{H}_{a})-\frac{4{\pi }^{2}}{90}\gamma {M}_{s}({\varepsilon }_{r}+5){(\frac{df}{{c}_{0}})}^{2},$$

where *H*_*a*_ - anisotropy field, *ε*_*r*_ - relative permittivity, *d* - sphere diameter, *c*_0_ - speed of electromagnetic wave in vacuum. It is assumed that *ε*_*r*_ = 16 for all the studied spheres^14^. The quadratic term in Eq. (5) may be owed to a size effect relative to the wavelength. In principle, additional terms of order higher than 2 can be added to Eq. (5) for improved accuracy. Eq. (5) is in good agreement with rigorous numerical MPR computations until the diameter of the sphere becomes comparable with the free-space electromagnetic wavelength^14^.

## Intrinsic vs Extrinsic Linewidth

In principle, if intrinsic properties of the ferromagnetic material are of interest, the conditions for driving the MPR resonance enforced inside the material, and not in its neighbourhood, should be considered. For that fundamental reason, the authors find it necessary to elucidate the relationship between the extrinsic (Δ*H*_*ext*_) and intrinsic (Δ*H*_*int*_) ferromagnetic linewidths. For spheres with diameter small as compared to the wavelength, the *f* ^2^ term in Eq. (5) can be dropped, leaving

6 $$f=\gamma ({H}_{ext}+{H}_{a}).$$

Alternatively, the resonant frequency *f* can be related to the intrinsic magnetic field bias

7 $$f=\gamma ({H}_{int}+\frac{{M}_{s}}{3}),$$

owing to the fact that the static magnetic field inside an ideal sphere is related to the external field by^7^:

8 $${H}_{int}={H}_{ext}-\frac{{M}_{s}}{3}+{H}_{a}.$$

The determination of the ferromagnetic 3 dB bandwidth Δ*f* can proceed essentially in two ways: as a parameter of a fit to the complex transmission spectrum^15,16^ or from the measured loaded *Q*-factor^17^:

9 $$Q=\frac{f}{{\rm{\Delta }}f},$$

which contains information about various losses of the measured setup, including coupling losses, conduction losses and radiation losses. Depending on the experimental conditions, the unloaded *Q*-factor can be de-embedded in order to determine intrinsic magnetic losses of the sample. Assuming that the magnetic anisotropy field *H*_*a*_ is frequency-independent, which is a good approximation for ferromagnetic materials as far as *f* ≫ *γH*_*a*_^18^, Δ*f* can be converted into the magnetic field domain using Eq. (6):

10 $${\rm{\Delta }}{H}_{ext}=\frac{{\rm{\Delta }}f}{\gamma }.$$

Combining Eqs (9) and (10) yields:

11 $${\rm{\Delta }}{H}_{ext}=\frac{f}{\gamma Q}.$$

In the literature there is a general understanding that the Gilbert damping factor is inversely proportional to the unloaded *Q*-factor^19,20^. It is in fact equal to half of the inverse of the *Q*-factor:

12 $$\alpha =\frac{1}{2Q}.$$

From Eqs (11) and (12) one can arrive at

13 $${\rm{\Delta }}{H}_{ext}=\frac{2\alpha }{\gamma }f,$$

which is a standard expression for fitting Δ*H*_*ext*_. A constant term, known as inhomogeneous broadening (Δ*H*_0_), is usually added to obtain a better fit to experimental data:

14 $${\rm{\Delta }}{H}_{ext}=\frac{2\alpha }{\gamma }f+{\rm{\Delta }}{H}_{0}.$$

The Gilbert damping factor *α* is also treated as a constant in the fitting procedures. From Eqs (4) and (12) one can conclude that

15 $${\rm{\Delta }}{H}_{int}=\frac{{H}_{int}}{Q},$$

which is in agreement with the predictions of the MPR model^14^. Eventually, from Eqs (7) and (15) one can relate Δ*H*_*ext*_ and Δ*H*_*int*_ by:

16 $${\rm{\Delta }}{H}_{ext}={\rm{\Delta }}{H}_{int}+\frac{{M}_{s}}{3Q},$$

or, equivalently^14^:

17 $${\rm{\Delta }}{H}_{ext}={\rm{\Delta }}{H}_{int}(1+\frac{{M}_{s}}{3{H}_{int}}).$$

As it can be seen, for a given internal field *H*_*int*_, Δ*H*_*int*_ depends solely on the *Q*-factor, whereas Δ*H*_*ext*_ is additionally implicitly dependent on the saturation magnetisation *M*_*s*_. In addition, a static demagnetisation factor $\frac{1}{3}$ of a sphere occurring in Eq. (17) indicates that Δ*H*_*ext*_ is geometry-dependent, i.e. it cannot be treated as the unequivocal material parameter. From this perspective Δ*H*_*int*_ is an intrinsic parameter of the material, and not Δ*H*_*ext*_. According to the authors’ knowledge, the spectral properties of Δ*H*_*int*_ of spherical samples have not been studied so far and therefore constitute a major goal of this article.

## Experiments

### Experimental setup and procedure

**Figure 1:**
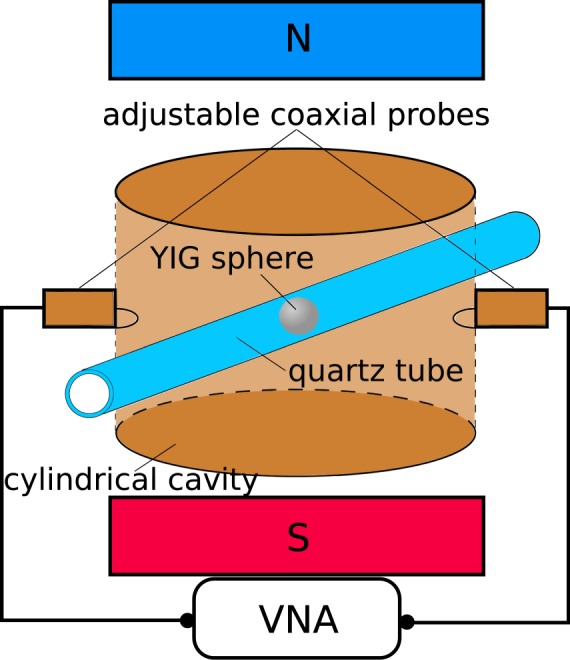
Schematic of the experimental setup. The cylindrical cavity, whose diameter is 6 mm and height is 5 mm, is loaded with the sample and placed between the electromagnet’s pole pieces. The quartz rod serves as support for the sample. The vector network analyzer (VNA) is coupled to the cavity by means of coaxial probes with loops whose insertion depth is adjustable. Drawing not to scale.

Measurements of the *Q*-factor vs. magnetic bias were performed in the setup shown schematically in Fig. 1, consisting of a brass subwavelength cylindrical cavity loaded with the spherical sample, an electromagnet and a vector network analyzer (VNA). The electromagnet and the VNA were controlled via a PC station. The measurement of a single YIG sphere proceeded as described in the following. The sphere was placed in a quartz tube of a 3 mm outer diameter. The tube was inserted into a cylindrical cavity of 6 mm in diameter and 5 mm in height in such a way that the sphere was located roughly in its geometric centre. The sphere was sandwiched between two styrofoam pieces in a way that its movement along the tube was limited but it was still able to rotate freely as the inner diameter of the tube was slightly larger than 0.5 mm, which is the diameter of the largest sample. Coupling to the sphere was realized by means of coaxial probes ended with loops, whose insertion depth was adjustable. The loops were adjusted when necessary to maintain weak coupling, i.e. |*S*_21_| < −40 dB for all magnetic bias fields, so that coupling losses can be neglected. The adjustment of the coupling loops also had the effect of limiting electromagnetic (EM) field disturbances inevitably caused by their presence. The cavity was placed between the pole pieces of a commercial electron paramagnetic resonance (EPR) spectrometer, which acted only as a bias source. After performing microwave calibration to the plane of the connectors of the adjustable probes, the complex *S*_21_ transmission spectrum for a range of magnetic bias values was measured. The resonant frequency and *Q*-factor were obtained from the measured data using an in-house developed circle fitting algorithm^21,22^, the employment of which became indispensable at higher frequencies, since the resonant curves showed strong asymmetry due to the combined effect of coupling crosstalk and phase shift^23^. The algorithm provided an estimate of the standard deviation of the *Q*-factor, however estimating the uncertainty of the resonant frequency was not considered. A total of 10 YIG spheres were measured, with 9 from a single vendor. The diameters of the spheres ranged from 0.305 mm to 0.5 mm and the *M*_*s*_ values were equal to 875 G, 1300 G, 1780 G, as specified by the vendor. The spheres with *M*_*s*_ < 1780 G were doped with gallium. The optimal range for the coupling |*S*_21_| to the cavity was found to be between −45 dB and −50 dB. For such a weak coupling the value of the loaded *Q*-factor can be practically considered equal to the unloaded *Q*-factor^24^. Such a low-coupling technique lowers the experimental errors in the determination of the *Q*-factor compared with measurements at stronger coupling since the coupling coefficients do not need to be known. Otherwise their uncertainties have to be considered, increasing the overall measurement uncertainty of the *Q*-factor. For low magnetic bias values, the microwave power was appropriately reduced to avoid the influence of Suhl instability^25^ on the *Q*-factor. In order not to compromise the signal-to-noise ratio (SNR) in this range, the spectrum averaging factors were appropriately increased.

The fundamental mode of the cylindrical cavity is the TM_010_ mode whose resonant frequency is 38.3 GHz, however, the presence of the quartz tube caused the resonant frequency to occur at ca. 29 GHz, so the measurements around that frequency were omitted. The spheres were allowed to rotate freely in the quartz tube, and thus oriented themselves in such a way that their easy magnetisation axis was more or less aligned with the external magnetic field. The level of misalignment is reflected in the anisotropy field *H*_*a*_ obtained by simultaneously fitting Eq. (5) to the applied magnetic field *H*_*ext*_ and measured resonant frequency *f*, as provided in Table 1. The anisotropy field of YIG along the easy magnetisation axis [111] is given by ${H}_{a}=\frac{4}{3}\frac{|{K}_{1}|}{{M}_{s}}$, where *K*_1_ is the first order cubic anisotropy constant^26^. At a temperature of 295 K, *K*_1_ ≈ −610 J^3^m^−1^ for pure YIG and thus *H*_*a*_ ≈ 57.4 Oe^27^. This value barely changes with increasing gallium concentration, which lowers both |*K*_1_| and *M*_*s*_^27^. The relationship between the resonant frequency and magnetic field set on the electromagnet did not exactly follow equation Eq. (5), which can be quite well explained by adding a correction term *H*_*corr*_ to *H*_*ext*_:

18 $${H}_{corr}={k}_{1}{H}_{ext}+{k}_{2}{H}_{ext}^{2},$$

where *k*_1_ and *k*_2_ were treated as fit parameters. Admittedly, *H*_*corr*_ turned out to vary from sphere to sphere, from ca. 0.3% to 0.9% of *H*_*ext*_, with larger spheres exhibiting a larger deviation, which cannot be explained by an error in the applied magnetic field alone. As previously analysed^8^, the presence of a metal shielding around the sphere can alter the resonant frequency if its radius becomes comparable with the radius of the sphere, however discrepancies from Eq. (5) amounted to less than 0.1% as computed using the MPR characteristic equation^7^ for the experimental conditions. Other factors that may have influenced the fitted value of *H*_*corr*_ are an inhomogeneous filling of the cavity or variations in the permittivity and saturation magnetization of the samples, which are challenging to identify precisely. Since the aforementioned size dependence of *H*_*corr*_ is not significant, it is assumed in this paper that the external magnetic field which acted on the sphere is *H*_*ext*_ + *H*_*corr*_.

**Table 1:** Summary of measurement and data processing results.

*M*_*s*_ (*G*)	*d* (mm)	*H*_*a*_ (Oe)	*a*	*b* (Oe)	${{\boldsymbol{R}}}_{{\boldsymbol{adj}}}^{{\bf{2}}}$	Vendor
1780	0.305	55.25	8.071 (57) × 10^−5^	0.0574 (15)	0.9984	#1
1780	0.381	55.61	1.0874 (99) × 10^−4^	0.0458 (19)	0.9976	#1
1780	0.483	56.13	9.691 (61) × 10^−5^	0.0590 (11)	0.9988	#1
1780	0.5	50.49	8.933 (68) × 10^−5^	0.1642 (20)	0.9896	#2
1300	0.305	54.68	2.229 (16) × 10^−5^	0.0564 (24)	0.9983	#1
1300	0.406	55.85	2.197 (21) × 10^−4^	0.0721 (28)	0.9973	#1
1300	0.483	58.79	2.376 (17) × 10^−4^	0.0493 (19)	0.9985	#1
875	0.305	58.74	2.383 (24) × 10^−4^	0.1337 (31)	0.9974	#1
875	0.381	57.59	2.391 (39) × 10^−4^	0.0637 (32)	0.9934	#1
875	0.483	59.93	2.371 (16) × 10^−4^	0.1018 (34)	0.9988	#1

*M*_*s*_ - saturation magnetisation, *d* - diameter, *H*_*a*_ - anisotropy field, *a* - slope of Δ*H*_*int*_(*H*_*int*_), *b* - intercept of Δ*H*_*int*_(*H*_*int*_), ${R}_{adj}^{2}$ - adjusted coefficient of determination. Theoretical values of *H*_*a*_ amount to ca. 58 Oe for each sample.

## Results

### Quality factor

**Figure 2:**
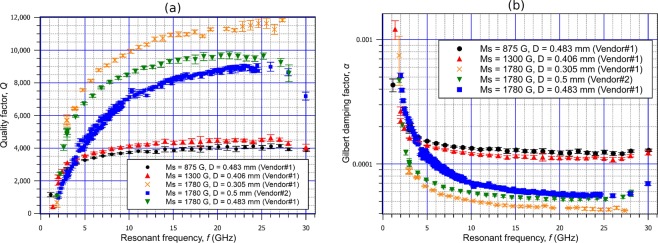
Measured *Q*-factor (**a**) and Gilbert damping factor *α* (**b**) of chosen spheres as a function of frequency. The relationship between *Q* and *α* is given by *α* = 1/(2*Q*) (Eq. 12).

The measured *Q*-factors vs. frequency for a few of the studied spheres are provided in Fig. 2. All curves behave in qualitatively the same manner. At first, the *Q*-factor increases with resonant frequency, and then stabilizes at a magnetic bias field that is well above the saturation magnetisation. In view of Eq. 12, *α* exhibits an inverse behaviour, i.e. drops quite rapidly until it reaches saturation. In our experiments damping can therefore be considered practically constant above a certain frequency that is not known *a priori*. This frequency does not necessarily increase with increasing *M*_*s*_. The increase of *α* in weak magnetic fields is a manifestation of the appearance of a magnetic domain structure^28^. The obtained dependence of *α* vs. frequency bears resemblance to reported spectra of *α* in ferromagnetic metals^29^, alloys^30^ and semiconductors^31^ measured using the time resolved magneto-optic Kerr effect (TRMOKE) technique in the microwave frequency range.

The brass cavity enclosure of the measured spheres is intended to suppress radiation losses, so the drop of the *Q*-factor above ca. 26 GHz visible in all spheres to a lesser or greater extent should rather be attributed to calibration errors or coupling losses. It has been experimentally determined that there is no contribution of radiation losses via the holes made for the quartz tube insertion that would exceed the uncertainty of the measured *Q*-factor at least up to 26 GHz. To confirm that, the quartz tube was shortened so that it would completely fit into the cavity. The entry holes were subsequently sealed shut with aluminium foil. Besides, it can be expected that radiation losses, if somehow present, cannot be significant since the cutoff frequency of entry holes of diameter 3 mm fully filled with quartz (*ε*_*r*_ = 3.8) is equal to 30 GHz. The tubes used in the described experiments were hollow, which ought to further increase the cutoff frequency.

### Measured intrinsic and extrinsic linewidths

**Figure 3:**
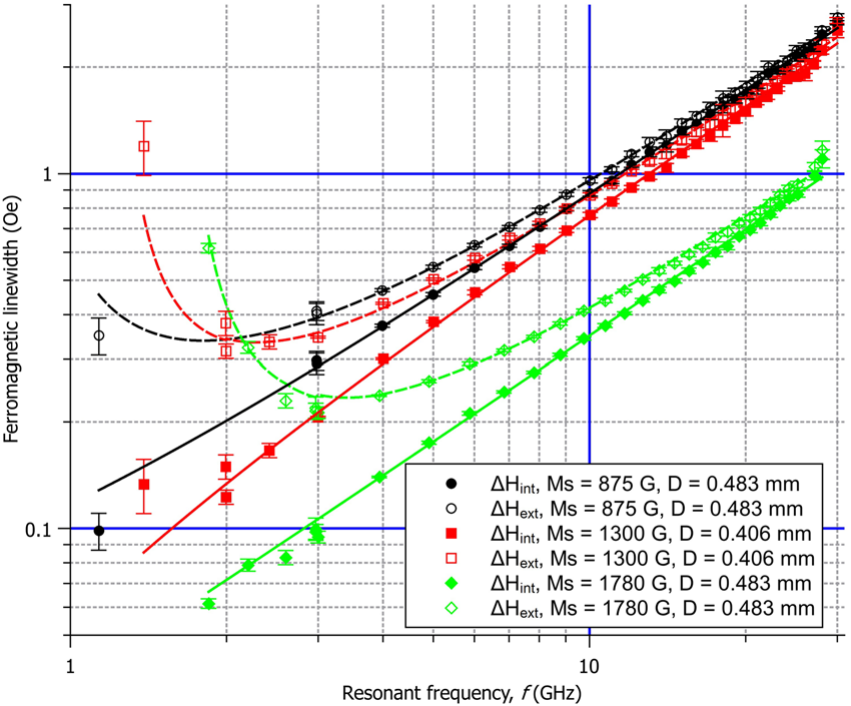
Comparison between intrinsic and extrinsic linewidths for chosen spheres of different values of the saturation magnetisation *M*_*s*_. Linear weighted least squares fits to the intrinsic linewidths Δ*H*_*int*_ are plotted in solid lines (−), and Eq. (17) is shown in dashed lines (−), calculated using the fitted Δ*H*_*int*_ values.

Based on the obtained *Q*, *f* and *H*_*int*_, Δ*H*_*ext*_ was computed using Eq. (11) and Δ*H*_*int*_ using Eq. (15). Both quantities are plotted vs. *f* in Fig. 3 for three spheres of different *M*_*s*_. As it is well-known from the literature^32^, *H*_*ext*_ does not follow Eq. (14) in the whole frequency range if one assumes a constant *α*. However, we find that Δ*H*_*int*_ does have a linear dependence on *f* in practically the whole range, as it can be seen in Fig. 3. The increase of Δ*H*_*ext*_ for low frequencies can be seen as a consequence of the increase of the damping factor *α* (compare Eq. (13)), but also as a geometric effect (compare Eq. (17)). Experiments confirm Eq. (17) in the measured frequency range, which show that Δ*H*_*ext*_ asymptotically converges to Δ*H*_*int*_. It should be stressed that although Δ*H*_*ext*_ approaches Δ*H*_*int*_ for large frequencies, the differences exceed the measurement uncertainties of the applied method. From Eq. (17) it follows that even at 30 GHz and for *M*_*s*_ = 1780 G, Δ*H*_*ext*_ is ca. 6% higher than Δ*H*_*int*_, while at 20 GHz this overestimation amounts to ca. 9%. Poor fitting of the linear model given by Eq. (14) to the experimental *ΔH*_*ext*_ values for *f* > 20 GHz is clearly visible in the distribution of the fit residuals. One can presume that this usually goes unnoticed due to the high experimental errors in most broadband FMR experiments^32^. Moreover, for high enough frequencies Eq. (14) is no longer valid in accordance with Eq. (5) due to the size effect. Nevertheless, in our experiments the relationship between Δ*H*_*int*_ and *f* has been found to be highly linear. For most of the spheres negative values of the intercept have been obtained, which is considered unphysical in the literature^33–35^. However, this can be seen as a yet another geometric effect. Combining Eqs (7), (12) and (15) yields:

19 $${\rm{\Delta }}{H}_{int}=2\alpha {H}_{int}=2\alpha (\frac{f}{\gamma }-\frac{{M}_{s}}{3}).$$

**Figure 4:**
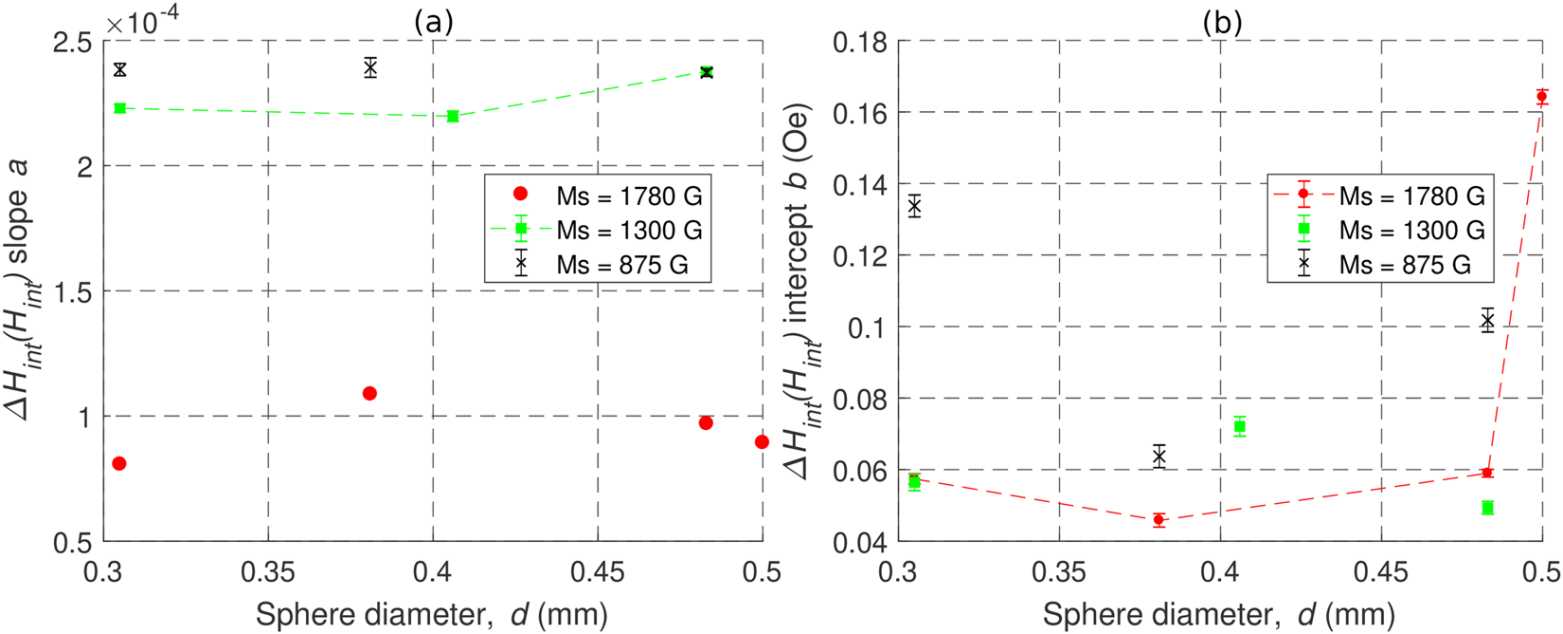
Slope (**a**) and intercept (**b**) of the linear relationship between the intrinsic ferromagnetic linewidth Δ*H*_*int*_ and the internal magnetic field *H*_*int*_ as a function of sphere diameter *d*. Results for a total of 10 samples are shown.

It can be noticed that when Δ*H*_*int*_ is plotted vs. *f*, it is decreased by $\frac{2\alpha {M}_{s}}{3}$. For all the aforementioned reasons, in this work the results of the analysis of Δ*H*_*int*_(*H*_*int*_) are reported. Fitting of the measurement results in the whole frequency bandwidth with a linear model Δ*H*_*int*_ = *aH*_*int*_ + *b* resulted in adjusted coefficients of determination ${R}_{adj}^{2}$ > 0.99 for all the studied spheres. The parameters of the fitted lines are summarized in Table 1, together with the coefficients of determination ${R}_{adj}^{2}$. The slopes and intercepts of the fitted lines have been additionally plotted separately as a function of the sphere diameter in Fig. 4.

### Assessment of conduction losses

The contribution of the conduction losses has been approximated in the way described in the following. Since there is no electrodynamic model of a spherical gyromagnetic resonator placed in a cylindrical cavity, in all calculations it is assumed that the sample is placed at the centre of a spherical cavity inscribed in the cylindrical cavity used in the experiments (see Fig. 1), having therefore a radius of 2.5 mm. The change of the cavity shape is not expected to lead to an underestimation of the conduction losses because such losses in shielded dielectric resonators occur in the part of the shielding that is the closest to the resonator. The proposed computational model should thus provide an upper limit for the conduction losses. Based on the measured Δ*H*_*int*_ values, the resonant frequency dependence of the intrinsic *Q*-factor (*Q*_*m*_) of each sample was calculated using the MPR characteristic equation^7^. The value of *Q*_*m*_ depends only on the magnetic losses of the sample according to Eq. (15). The *Q*-factor associated with the losses in cavity walls (*Q*_*c*_) was computed using the incremental frequency rule^14,36^. The conductivity of the spherical cavity walls is assumed to be *σ* = 1.67 × 10^7^ S m^−1^, which is ca. 28% of the conductivity of copper and is a typical value for brass. The total *Q*-factor is computed as:

20 $$Q={({Q}_{m}^{-1}+{Q}_{c}^{-1})}^{-1}.$$

**Figure 5:**
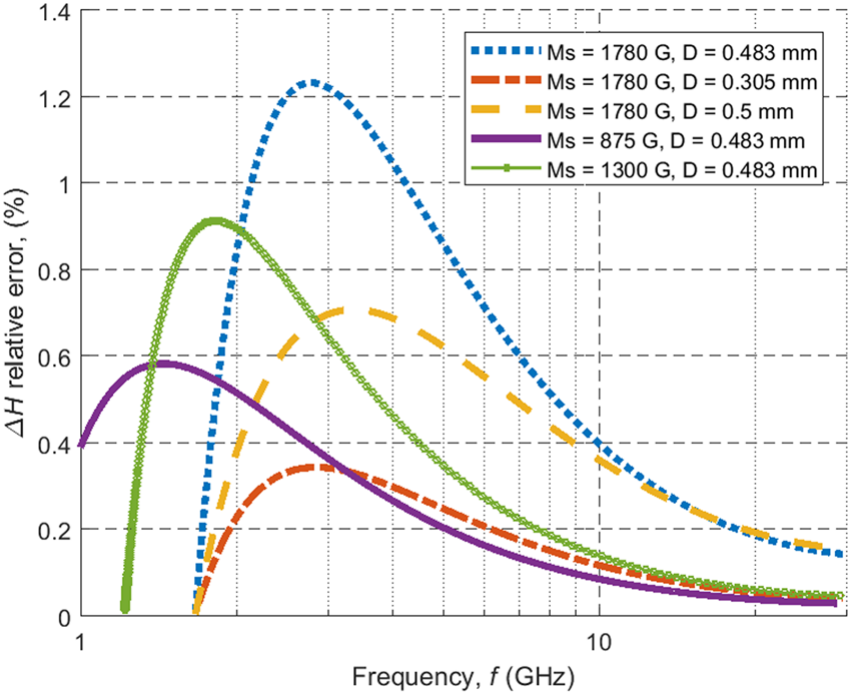
Estimated relative error in the linewidth for chosen spheres (see figure legend) due to conductor losses in the metal surrounding the sample as calculated using the magnetic plasmon resonance (MPR) characteristic equation. The error in the linewidth is small, which is understood since the sphere concentrates the bulk of the electromagnetic (EM) energy of the system^8^.

Figure 5 depicts the relative error in Δ*H*_*int*_ due to conductor losses:

21 $$\delta ({\rm{\Delta }}{H}_{int})=\frac{\frac{{H}_{int}}{Q}-\frac{{H}_{int}}{{Q}_{m}}}{\frac{{H}_{int}}{{Q}_{m}}}=\frac{{Q}_{m}}{{Q}_{c}},$$

vs. resonant frequency. As it can be seen the error in the determination of the linewidth is equal to the ratio *Q*_*m*_/*Q*_*c*_. As shown in Fig. 5 the theoretical error is typically far below 1% for the measured samples and can therefore be neglected. The value of *Q*_*c*_ is much larger than *Q*_*m*_ due to the fact that the sphere supports the MPR mode and the EM field outside of the sample is evanescent^7^.

## Discussion and Conclusions

In this work, a method enabling the determination of the intrinsic ferromagnetic linewidth (Δ*H*_*int*_) of the monocrystalline spheres from the accurate broadband measurements of the *Q*-factor has been demonstrated. A new variation of known techniques for measuring the *Q*-factor of garnet spheres was used, in which the coupling was weak, thus, reducing experimental errors. Non-magnetic losses have been assessed using the MPR model. The results have been used to elucidate the relationship between the extrinsic (Δ*H*_*ext*_) and intrinsic (Δ*H*_*int*_) ferromagnetic linewidths. The latter quantity has been found to have a linear dependence on the internal magnetic field, with the coefficient of determination ${R}_{adj}^{2}$> 0.99 and positive intercept (b) values. It is therefore possible to characterize the magnetic loss of the studied monocrystalline spheres up to mm-wave frequencies using only two parameters. Namely, the slope (a) and intercept (b) of the Δ*H*_*int*_ vs. *H*_*int*_ dependence. Nonetheless, the authors wish to stress that this may not be a property common to all monocrystalline YIG spheres due to the influence of various factors, such as surface roughness or porosity, on Δ*H*_*int*_. In particular, it does not hold at low frequencies for polycrystalline spheres which may exhibit the Buffler peak^37^. Moreover, another major novel conclusion that can be drawn from this paper is that the high-frequency estimate of the Gilbert damping factor *α*, which is frequently sought in the literature, can be obtained from low-frequency measurements of Δ*H*_*int*_. Obtaining this parameter from Δ*H*_*ext*_ data is inherently burdened by a large systematic error that can be viewed as a geometric effect resulting from relating Δ*H*_*ext*_ to external magnetic bias. The authors hope that this work will further convince the scientific community to incorporate the MPR into their studies on ferromagnetic spheres and be a step towards the standardization of the proposed straightforward method for the determination of the intrinsic ferromagnetic linewidth by direct *Q*-factor measurement.
